# A novel mouse model of anterior segment dysgenesis (ASD): conditional deletion of *Tsc1* disrupts ciliary body and iris development

**DOI:** 10.1242/dmm.028605

**Published:** 2017-03-01

**Authors:** Anna-Carin Hägglund, Iwan Jones, Leif Carlsson

**Affiliations:** Umeå Center for Molecular Medicine (UCMM), Umeå University, Umeå 901 87, Sweden

**Keywords:** *Tsc1*, mTORC1, Pax6, Ciliary body, Iris, Anterior segment dysgenesis

## Abstract

Development of the cornea, lens, ciliary body and iris within the anterior segment of the eye involves coordinated interaction between cells originating from the ciliary margin of the optic cup, the overlying periocular mesenchyme and the lens epithelium. Anterior segment dysgenesis (ASD) encompasses a spectrum of developmental syndromes that affect these anterior segment tissues. ASD conditions arise as a result of dominantly inherited genetic mutations and result in both ocular-specific and systemic forms of dysgenesis that are best exemplified by aniridia and Axenfeld–Rieger syndrome, respectively. Extensive clinical overlap in disease presentation amongst ASD syndromes creates challenges for correct diagnosis and classification. The use of animal models has therefore proved to be a robust approach for unravelling this complex genotypic and phenotypic heterogeneity. However, despite these successes, it is clear that additional genes that underlie several ASD syndromes remain unidentified. Here, we report the characterisation of a novel mouse model of ASD. Conditional deletion of *Tsc1* during eye development leads to a premature upregulation of mTORC1 activity within the ciliary margin, periocular mesenchyme and lens epithelium. This aberrant mTORC1 signalling within the ciliary margin in particular leads to a reduction in the number of cells that express Pax6, *Bmp4* and *Msx1*. Sustained mTORC1 signalling also induces a decrease in ciliary margin progenitor cell proliferation and a consequent failure of ciliary body and iris development in postnatal animals. Our study therefore identifies *Tsc1* as a novel candidate ASD gene. Furthermore, the *Tsc1*-ablated mouse model also provides a valuable resource for future studies concerning the molecular mechanisms underlying ASD and acts as a platform for evaluating therapeutic approaches for the treatment of visual disorders.

## Abstract (precis)

**Summary:** Conditional deletion of *Tsc1* in the developing eye results in ciliary body and iris hypotrophy, demonstrating that aberrant mTORC1 signalling contributes to anterior segment dysgenesis (ASD).

## INTRODUCTION

Anterior segment dysgenesis (ASD) encompasses a spectrum of autosomal dominant developmental syndromes that affect tissues within the anterior segment of the eye including the cornea, lens, iris, ciliary body (CB) and associated drainage structures involving the trabecular meshwork (TM) and Schlemm's canal. Development of these tissues involves a complex interplay between neuroectodermal cells originating from the ciliary margin (CM) of the optic cup, neural crest cells residing within the overlying periocular mesenchyme (POM) and ectodermal lens epithelial (LE) cells. ASD is caused by genetic mutations that affect these cellular domains and presents as either autonomous ocular anomalies or ocular anomalies accompanied by systemic deficits. Ocular-specific ASD is a group of syndromes that solely affect the eye and its associated structures. This group is best exemplified by aniridia which is a neuroectoderm-derived panocular disorder that is associated predominantly with mutations in paired box gene 6 (*PAX6*). Individuals affected by aniridia present with distinct anterior segment deficits such as iris hypoplasia and cataracts. By contrast, the systemic forms of ASD are a group of closely related diseases with Axenfeld–Rieger syndrome (ARS) and Peter's anomaly (PA) being amongst the best characterised. Both are neural crest-derived disorders and result predominantly from mutations in either paired-like homeodomain 2 (*PITX2*) or forkhead box C1 (*FOXC1*). ARS and PA share common and highly penetrant anterior segment deficits that primarily affect the pupil and drainage structures (Cvekl and Tamm, 2004; Gould and John, 2002; Graw, 2010; Idrees et al., 2006; Ito and Walter, 2014; Lee et al., 2008; Reis and Semina, 2011; Sowden, 2007).

Diagnosis of the precise ASD syndrome is challenging because of the extensive clinical overlap observed amongst related diseases. The generation of animal models has therefore proved to be an effective approach for deciphering the molecular aetiology underlying this heterogeneity (Gould and John, 2002). Haploinsufficiency in the mouse *Pax6* gene results in a clinical model of aniridia with iris hypoplasia being prevalent; while modulation of *Pitx2* and *Foxc1* gene dose results in pupil and drainage structure abnormalities and thus represent experimental models of ARS (Baulmann et al., 2002; Gage et al., 1999; Hill et al., 1991; Hogan et al., 1988; Holmberg et al., 2004; Lines et al., 2002; Ramaesh et al., 2003; Smith et al., 2000). However, the severity of ASD presentation for a given mutation is highly dependent on genetic background (Chang et al., 2001; Mao et al., 2015; Smith et al., 2000). These combined observations therefore demonstrate that ASD presents as a complex spectrum of phenotypes and that modifier genes influence the severity of disease presentation. Furthermore, it is clear that additional genes underlying several ASD syndromes remain unidentified. Identification and characterisation of these unknown disease-causing genes using animal models will therefore facilitate an integrated understanding of the pathogenic mechanisms involved in ASD. Moreover, it is entirely possible that these unknown ASD-causing genes could be the underlying genetic basis for other systemic disorders that present with eye involvement (Gould and John, 2002; Reis and Semina, 2011; Sowden, 2007).

Tuberous sclerosis complex (TSC) is a systemic syndrome that is caused by inactivating point mutations in either hamartin (*TSC1*) or tuberin (*TSC2*), which leads to sustained activation of the mTORC1 signalling pathway and consequent formation of tumour-like lesions (referred to as hamartomas) in affected organs (Han and Sahin, 2011; Napolioni et al., 2009). TSC patients exhibit complex neurological deficits and half of all affected individuals present with visual system involvement that is best characterised by the presence of hamartomas within the posterior segment of the eye (Mennel et al., 2007; Samueli et al., 2015). However, in isolated cases, some TSC patients also present with anterior segment deficiencies, which suggests that this systemic syndrome also involves ASD in individual cases (Eagle et al., 2000; Gutman et al., 1982; Kranias and Romano, 1977; Lucchese and Goldberg, 1981; Milea and Burillon, 1997; Welge-Lussen and Latta, 1976).

The generation and characterisation of an eye-specific TSC mouse model that recapitulated the classic neuropathological hallmarks of this multiorgan syndrome was recently reported (Jones et al., 2015). That study provided the first major insight into the molecular aetiology of TSC within the posterior segment of the eye and demonstrated a pivotal role for *Tsc1* in regulating various aspects of visual pathway development. The work presented in this current report also identifies *Tsc1* as a novel ASD candidate gene since ablation of *Tsc1* during eye development leads to CB and iris hypoplasia within the anterior segment of the eye. This novel mouse model therefore provides a valuable resource for future studies concerning the molecular mechanisms underlying eye development in addition to serving as a platform to evaluate new therapeutic approaches for the treatment of visual disorders.

## RESULTS

### Eye-specific conditional deletion of *Tsc1* using a novel Cre-Lox system

**Fig. 1.**
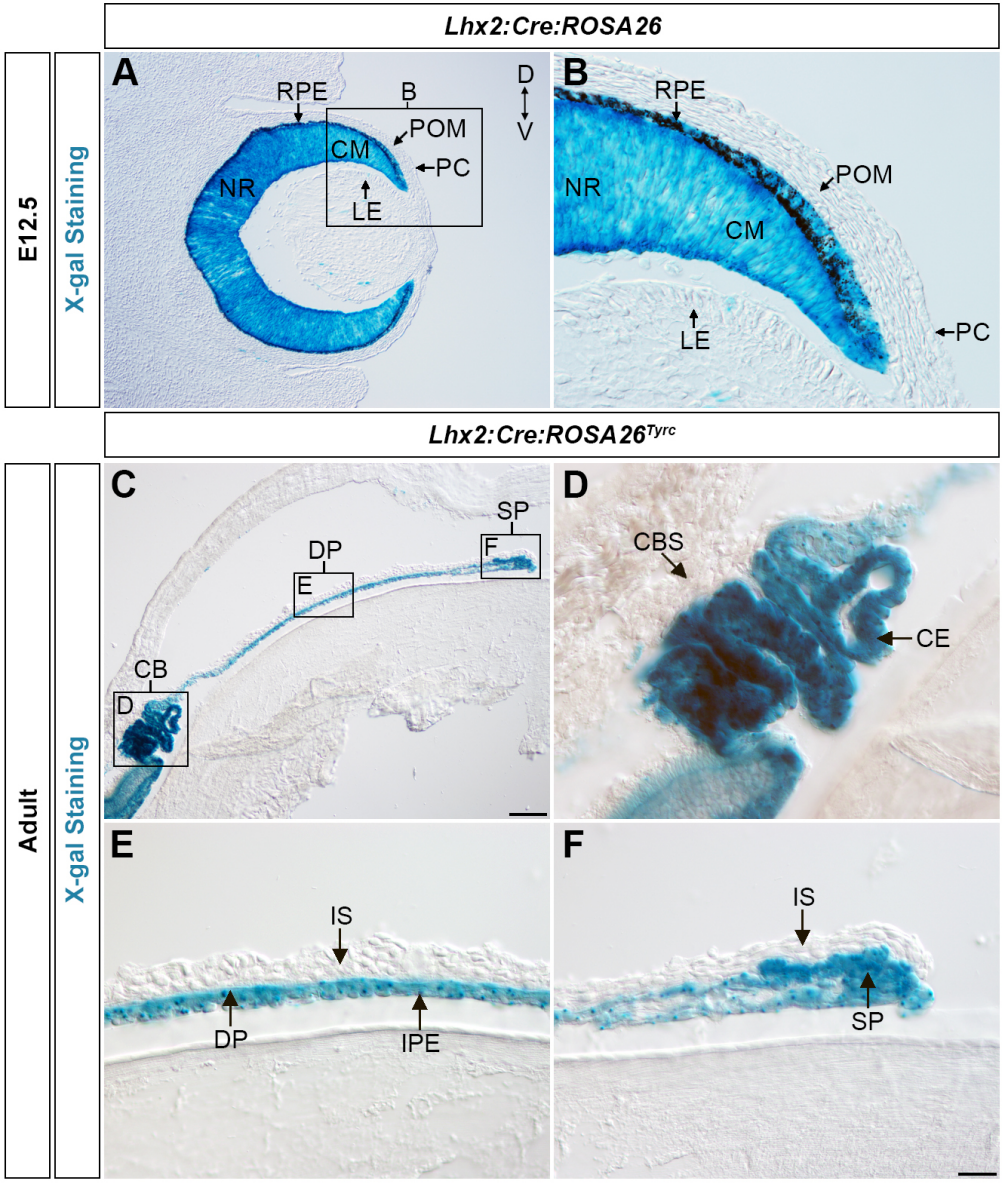
**Lineage-tracing analysis of Cre recombinase expression in *Lhx2-Cre:ROSA26R* and *Lhx2-Cre:ROSA26R^Tyrc^* mice.** (A,B) X-gal staining in the developing eye of an *Lhx2-Cre:ROSA26R* mouse at E12.5 demonstrating β-gal activity in the NR, CM and RPE and confirming Cre-recombinase expression in these domains. No β-gal activity is observed in the LE, overlying POM or PC because of a lack of Cre recombinase expression in these regions. (C) X-gal staining of the anterior eye segment from an adult *Lhx2-Cre*:*ROSA26R^Tyrc^* mouse demonstrating β-gal activity in the CB and iris. (D) X-gal staining of the CB from a *Lhx2-Cre*:*ROSA26R^Tyrc^* mouse demonstrating β-gal activity in the CE. (E) X-gal staining of the medial iris from a *Lhx2-Cre:ROSA26R^Tyrc^* mouse, demonstrating β-gal activity exclusively in the DP and IPE. (F) X-gal staining of the distal iris tip from a *Lhx2-Cre*:*ROSA26R^Tyrc^* mouse demonstrating β-gal activity in the SP. No β-gal activity is observed in the IS because of the lack of Cre-recombinase expression in this tissue. All adult *Lhx2-Cre*:*ROSA26R^Tyrc^* mice were analysed when older than 6 weeks. Scale bars: 100 µm (A,C), 25 µm (B,D-F). CB, ciliary body; CBS, ciliary body stroma; CE, ciliary epithelium; CM, ciliary margin; D, dorsal; DP, dilator pupillae; IPE, iris pigment epithelium; IS, iris stroma; LE, lens epithelium; NR, neural retina; PC, prospective corneal ectoderm; POM, periocular mesenchyme; RPE, retinal pigment epithelium; SP, sphincter pupillae; V, ventral; X-gal, 5-bromo-4-chloro-3-indolyl-β-D-galactopyranoside.

The creation of an eye-specific *Tsc1* conditional mouse model (*Lhx2-Cre:Tsc1^f/f^*) was recently described (Jones et al., 2015). Lineage tracing analysis in *ROSA26R* mice (Soriano, 1999) demonstrated that the *Lhx2-Cre* transgene used to generate this model promotes recombination in progenitor cells that generate the CM (Fig. 1A,B). No *Lhx2-Cre* transgene expression was observed in the LE, overlying POM or prospective corneal ectoderm (PC) (Fig. 1A,B). Next, it was determined which anterior segment structures these lineage-traced CM cells contributed to. These experiments were conducted in adult *ROSA26R* mice that had been bred to be homozygous for the *Tyr^c^* mutation (referred to as *Lhx2-Cre*:*ROSA26R^Tyrc^*; see Materials and Methods) to prevent masking of β-galactosidase (β-gal) activity in pigmented cells (Fig. 1C). The lineage-traced CM cells were found to contribute to the ciliary epithelia (CE) of the CB (Fig. 1D), the iris pigment epithelium (IPE) (Fig. 1E) and both sets of iridial muscles; the dilator pupillae (DP) and the sphincter pupillae (SP), respectively (Fig. 1E,F). No lineage-traced CM cells were found to contribute to either the CB stroma (CBS) or iris stroma (IS) (Fig. 1D-F).

Immunohistochemical analysis was subsequently performed on adult *Lhx2-Cre*:*ROSA26R^Tyrc^* mice to independently confirm the lineage-tracing results. These analyses were conducted using established antibody markers to demarcate specific components of the anterior segment. Pax6 is expressed in the epithelial layers of both the CB and iris (Davis et al., 2009; Davis-Silberman et al., 2005) and the colocalisation of both β-gal^+^ and Pax6^+^ cells was detected in the CE (Fig. S1A-C) and IPE (Fig. S1D-F) of adult *Lhx2-Cre*:*ROSA26R^Tyrc^* mice, thus confirming transgene expression in the CM cells that generate these anterior segment tissues. Alpha smooth muscle actin (αSMA or ACTA2) is expressed in the iridial muscles (Davis et al., 2009; Davis-Silberman et al., 2005) and colocalisation of this protein and β-gal was detected in the DP (Fig. S1G-I) and SP (Fig. S1J-L), confirming that the *Lhx2-Cre* transgene is also expressed in CM cells that contribute to iridial muscle formation. Taken together, the lineage-tracing analyses demonstrated that the *Lhx2-Cre* transgene is a powerful molecular tool that can be used to elucidate the genetic networks that underlie both development and disease of the CE and IPE in addition to DP and SP.

### Conditional deletion of *Tsc1* during eye development leads to ASD

**Fig. 2.**
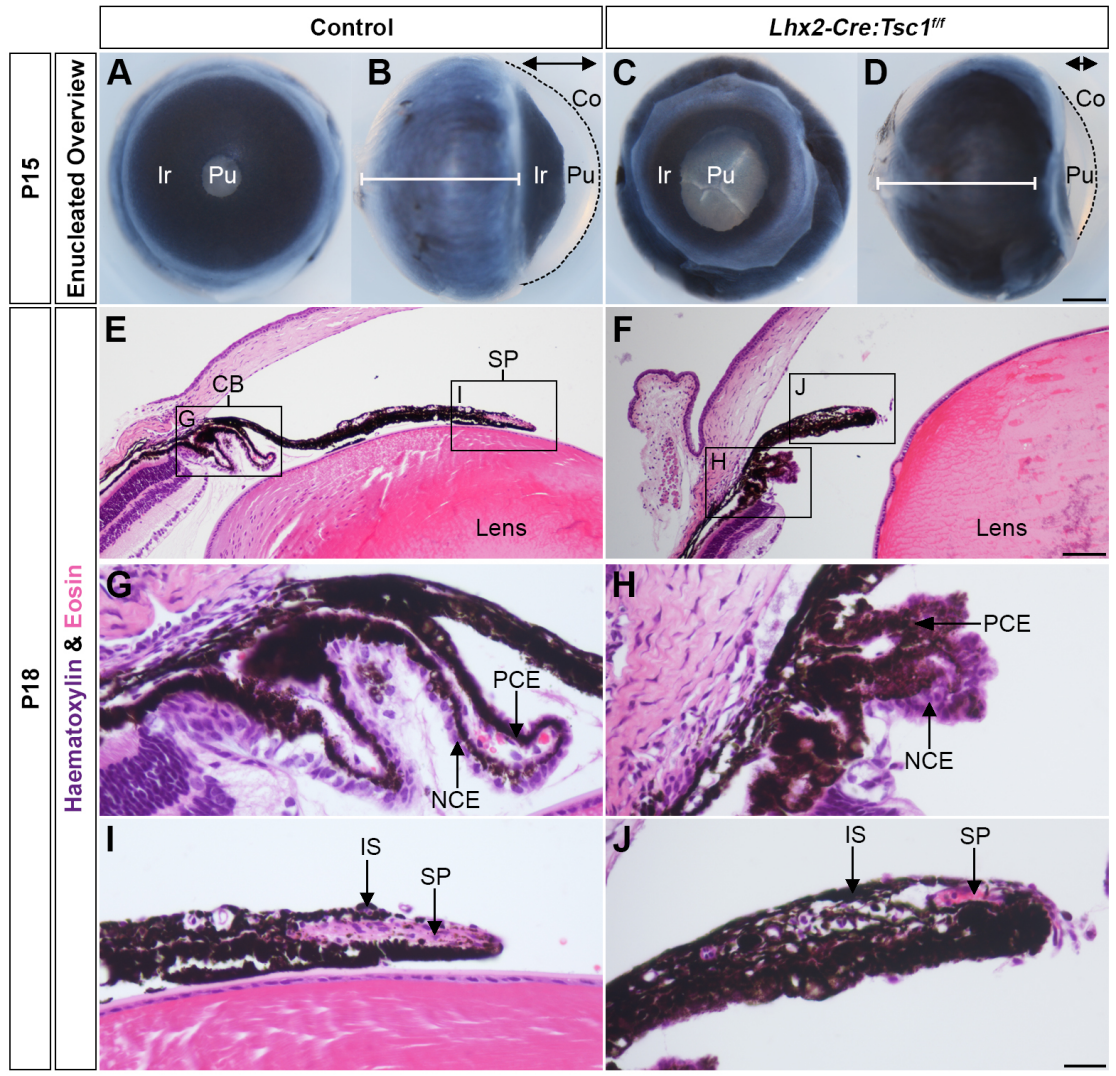
**Conditional deletion of *Tsc1* leads to ASD.** (A,C) Frontal and lateral (B,D) views of enucleated eyes taken from control (A,B) and mutant animals (C,D) at P15 showing severe iris hypoplasia and pupil enlargement in *Lhx2-Cre:Tsc1^f/f^* mice. Also evident in mutant mice is a reduction in anterior chamber volume (B,D, double arrow) and a shorter cornea with reduced curvature (B,D, broken line) when compared with control animals. *Lhx2-Cre:Tsc1^f/f^* mice also show greater posterior eye segment depth when compared with control animals (B,D, white lines). The line represents the depth of the posterior eye segment in control animals for direct comparison to *Lhx2-Cre:Tsc1^f/f^* mice. (E,F) Histological analysis of sagittal eye sections at P18, demonstrating an almost complete lack of CB structure and iris hypoplasia in *Lhx2-Cre:Tsc1^f/f^* mice. (G,H) Histological analysis of sagittal eye sections at P18. Although the epithelial layers of the CB are present, the overall CB structure within *Lhx2-Cre:Tsc1^f/f^* mice exhibits undefined ciliary processes. (I,J) Histological analysis of sagittal eye sections at P18 demonstrating SP atrophy in *Lhx2-Cre:Tsc1^f/f^* mice. Scale bars: 500 µm (A-D), 100 µm (E,F) and 50 µm (G-J). Co, cornea; Ir, iris; NCE, non-pigmented ciliary epithelium; PCE, pigmented ciliary epithelium; Pu, pupil.

The morphological appearance of the anterior eye segment in control and *Lhx2-Cre:Tsc1^f/f^* postnatal mice was first compared to elucidate the consequences of ablation of *Tsc1* within the CM (Fig. 2). The anterior segment of mutant mice had consistent morphological deficits that were fully penetrant on the *129/SvCBAC57BL/6* mixed genetic background (*n*=17 eyes). Iris hypoplasia was evident in frontal views of enucleated *Lhx2-Cre:Tsc1^f/f^* eyes at postnatal day (P)15 that resulted in a 2-fold enlargement of the centrally located pupil compared with control animals (Fig. 2A,C and Fig. S2). Moreover, lateral views of the enucleated eyes demonstrated that the anterior eye chamber in *Lhx2-Cre:Tsc1^f/f^* mice was reduced in volume (Fig. 2B,D, double arrows), with the cornea being shorter and exhibiting reduced curvature when compared with control littermates (Fig. 2B,D, black lines). However, the posterior eye segment of *Lhx2-Cre:Tsc1^f/f^* mice was larger than that of control animals, as previously documented (Fig. 2B,D, white lines) (Jones et al., 2015).

Histological analysis on sagittal eye sections at P18 (Fig. 2E-J) demonstrated that control mice exhibited a CB with well-defined ciliary processes and that the iris appeared to follow the curvature of the lens, with the SP evident at the distal tip (Fig. 2E). In contrast, both the CB and iris of mutant animals appeared hypotrophic with an apparent lack of ciliary processes and iris extension (Fig. 2F). Higher-magnification images confirmed that the CB of control animals was foliated and consisted of an ordered arrangement of both pigmented (PCE) and non-pigmented ciliary epithelia (NCE). In comparison, the overall CB structure in *Lhx2-Cre:Tsc1^f/f^* mice lacked any sign of well-defined ciliary processes with the PCE and NCE appearing to coalesce (Fig. 2H). Finally, higher-magnification images also revealed the SP of control animals as an elongated oval structure located in the distal iris tip (Fig. 2I). In contrast, the iris in *Lhx2-Cre:Tsc1^f/f^* mice exhibited a thickened club-like appearance with atrophic SP (Fig. 2J).

**Fig. 3.**
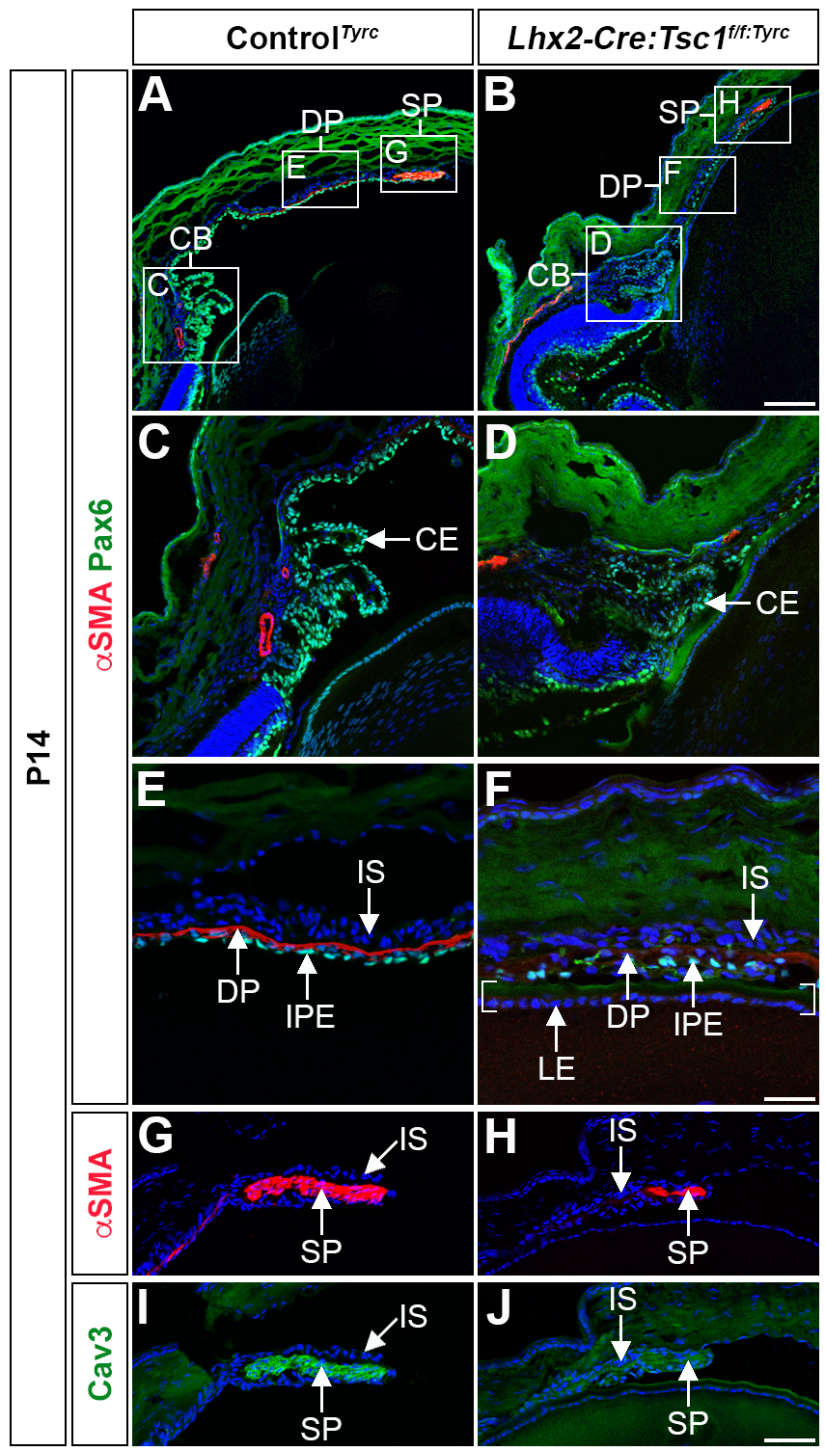
**Conditional deletion of *Tsc1* leads to ciliary body and iris hypoplasia.** (A,B) Immunohistochemical analyses of control*^Tyrc^* (A) and *Lhx2-Cre:Tsc1^f/f^*^:*Tyrc*^ mice (B) demonstrates atrophic anterior segment structures as defined by Pax6 and αSMA immunoreactivity. (C,D) Immunohistochemical analysis of the CB in control*^Tyrc^* (C) and *Lhx2-Cre:Tsc1^f/f^*^:*Tyrc*^ mice (D) shows a disorganised Pax6^+^ CE and a consequent loss of ciliary processes in mutant animals. (E,F) Immunohistochemical analysis of the medial iris in control*^Tyrc^* (E) and *Lhx2-Cre:Tsc1^f/f^*^:*Tyrc*^ mice (F) demonstrates irregularly positioned Pax6^+^ cells in the IPE of mutant animals. The medial iris of *Lhx2-Cre:Tsc1^f/f^*^:*Tyrc*^ mice also lacks a well-defined DP as demonstrated by a reduced level of αSMA^+^ immunoreactivity. In this image, the overlying IPE is in close proximity to the LE, which is defined by brackets. (G-J) Immunohistochemical analysis of the proximal iris in control*^Tyrc^* (G,I) and *Lhx2-Cre:Tsc1^f/f^*^:*Tyrc*^ mice (H,J) demonstrates that the SP in the mutant animals lacks its well-defined elongated shape and exhibits reduced levels of both αSMA (H) and Cav3 (J). All analyses were performed on mice at P14. Scale bars: 100 µm (A,B), 50 µm (C,D,G,H) and 25 µm (E,F).

Immunohistochemical analyses were performed at P14 to elucidate the underlying molecular aetiology for the hypotrophic appearance of the CB and iris in *Lhx2-Cre:Tsc1^f/f^* mice (Fig. 3). These experiments were conducted using antibody markers to demarcate specific structures within the anterior eye segment in mice homozygous for the *Tyr^c^* mutation (referred to as control*^Tyrc^* or *Lhx2-Cre:Tsc1^f/f:Tyrc^*; see Materials and Methods) to avoid the masking of antibody fluorescence in pigmented cells. Overview images showed the presence of Pax6^+^ cells in the anterior segment of both control*^Tyrc^* and *Lhx2-Cre:Tsc1^f/f:Tyrc^* mice (Fig. 3A,B). Higher-magnification analyses demonstrated that the CB of control animals was well defined, with Pax6^+^ CE cells organised as foliated ciliary processes (Fig. 3C). However, mutant animals exhibited a disorganised Pax6^+^ CE that resulted in indistinct ciliary processes (Fig. 3D). The IPE of control mice exhibited an ordered layer of Pax6^+^ cells but the equivalent epithelial layer in *Lhx2-Cre:Tsc1^f/f^* mice appeared irregular and disorganised (Fig. 3E,F). Additionally, although the presence of αSMA was detected in the iridial muscles (Fig. 3E-H), the levels of αSMA within *Lhx2-Cre:Tsc1^f/f^* mice was noticeably reduced, with the SP in particular being atrophic (Fig. 3G,H). To independently validate the observed SP atrophy, the expression of a mature SP-specific marker caveolin-3 (Cav3) was characterised (Kogo et al., 2006). Expression of Cav3 could not be detected within the SP of *Lhx2-Cre:Tsc1^f/f:Tyrc^* mice (Fig. 3I,J).

Taken together, these results demonstrate that conditional deletion of *Tsc1* within the CM during eye development leads to CB and iris hypoplasia in postnatal mice. It should be noted that in *Lhx2-Cre:Tsc1^f/f^* mice (*129/SvCBAC57BL/6* mixed genetic background, *n*=17 eyes), the CB and iris phenotype was fully penetrant and reproducibly severe in mutant animals. However, in the *Lhx2-Cre:Tsc1^f/f:Tyrc^* mice (*129/SvCBAC57BL/6NMRI* mixed genetic background, *n*=9 eyes) the severity of the anterior segment phenotype was variable among affected animals, albeit with full penetrance (Fig. S3). For example, although the CB in *Lhx2-Cre:Tsc1^f/f:Tyrc^* mice was always hypotrophic, the ciliary processes in some eyes were underdeveloped (Fig. S3F), whereas other eyes had ciliary processes that were barely detectable (Fig. S3R). Moreover, in some individuals, the iris appeared as a shortened structure completely lacking a DP (Fig. S3E,G) whereas other animals exhibited somewhat normal iris extension, albeit with obvious DP atrophy (Fig. S3M,O). Finally, the size and position of the SP was also variable in *Lhx2-Cre:Tsc1^f/f:Tyrc^* mice (Fig. S3H,L,P,T). It therefore appears that genetic background influences the presentation of the ASD phenotype observed in this study.

### Conditional deletion of *Tsc1* leads to elevation of mTORC1 signalling in tissues underlying ciliary body and iris development

Elevations in mTORC1 signalling within the CM following *Tsc1* ablation could cause the CB and iris phenotype observed in postnatal *Lhx2-Cre:Tsc1^f/f^* mice (Fig. S4A). Immunoblot analyses were therefore performed on eye homogenates at E14.5 to confirm conditional deletion of *Tsc1*. A significant reduction in the amount of hamartin (Tsc1) was detected in *Lhx2-Cre:Tsc1^f/f^* mice compared with control animals thus confirming eye-specific ablation of *Tsc1* (Fig. S4B,C). The level of phosphorylation of the S6 ribosomal protein Rps6 (pS6) was next assessed since this is an established marker for assessing upregulation of mTORC1 signalling following *Tsc1* ablation (Fig. S4A) (Biever et al., 2015; Kwiatkowski et al., 2002; Meikle et al., 2007). Significant increases in the levels of both pS6^S235/236^ and pS6^S240/244^ were observed in the eye homogenates of *Lhx2-Cre:Tsc1^f/f^* mice, thus confirming upregulation of mTORC1 signalling following *Tsc1* deletion (Fig. S4B,C).

**Fig. 4.**
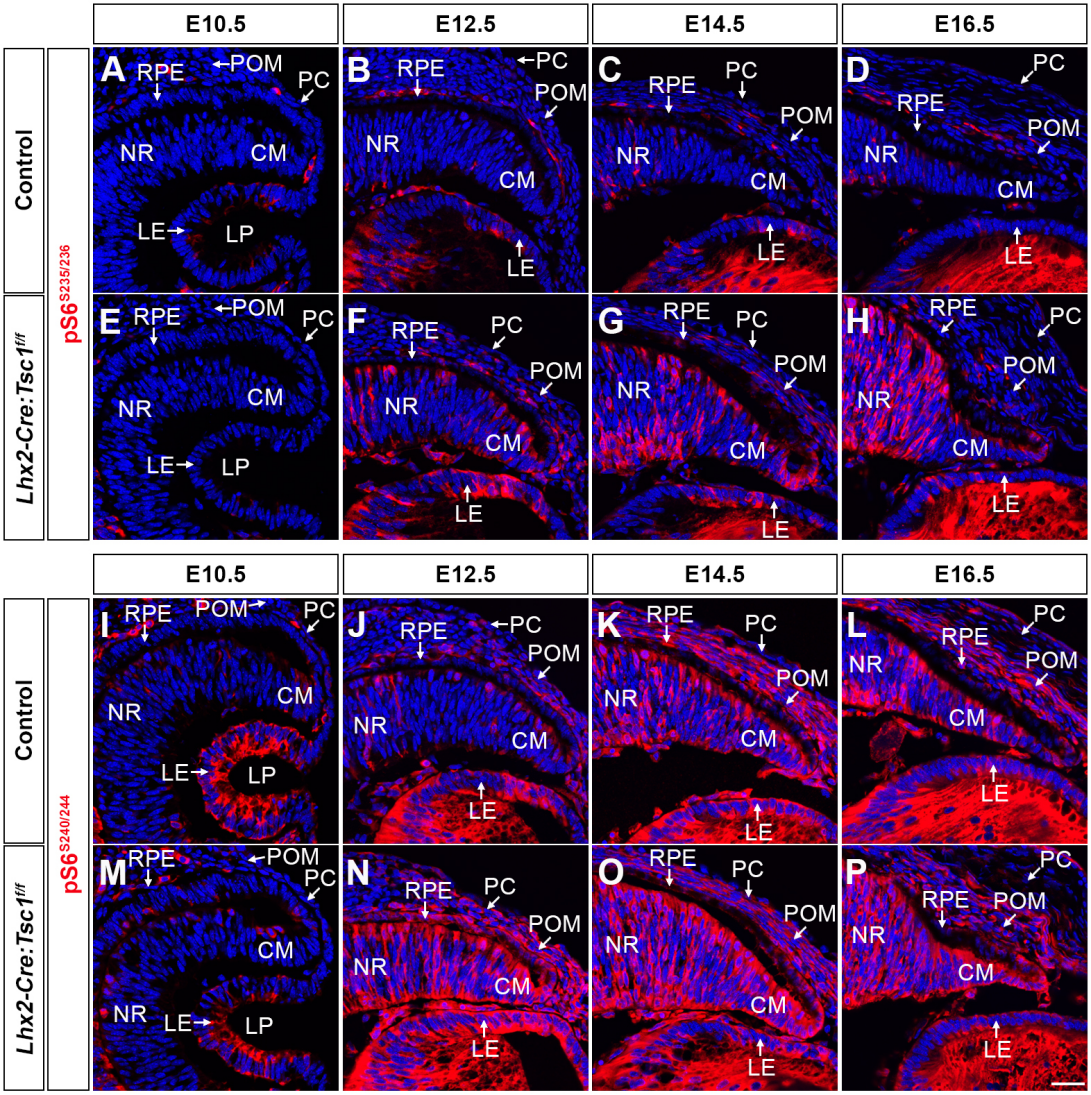
**Conditional deletion of *Tsc1* leads to an elevation of mTORC1 signalling in tissue domains that underlie anterior segment development.** (A-H) Immunohistochemical analysis of pS6^S235/236^ in coronal eye sections taken from control (A-D) and *Lhx2-Cre:Tsc1^f/f^* mice (E-H). No difference in pS6^S235/236^ activity is seen at E10.5 (A,E). A modest elevation of pS6^S235/236^ is detected within the CM, POM and LE of mutant animals at E12.5 (B,F). The levels of pS6^S235/236^ continue to be elevated at E14.5 (C,G) and E16.5 (D,H) within the CM of *Lhx2-Cre:Tsc1^f/f^* mice, whereas pS6^S235/236^ levels within the POM and LE are similar to controls. (I-P) Immunohistochemical analysis of pS6^S240/244^ in coronal eye sections taken from control (I-L) and *Lhx2-Cre:Tsc1^f/f^* mice (M-P). No difference in pS6^S240/244^ activity is seen at E10.5 (I,M). A robust elevation of pS6^S240/244^ is detected within the CM, POM and LE of mutant animals at E12.5 (J,N). The levels of pS6^S240/244^ continue to be elevated at E14.5 (K,O) and E16.5 (L,P) within the CM of *Lhx2-Cre:Tsc1^f/f^* mice, whereas pS6^S240/244^ levels within the POM and LE are similar to controls. Scale bar: 25 µm (A-P). LP, lens pit.

Development of the CB and iris in the mouse begins at E12.5 and involves interplay between cells residing in the CM, the overlying POM and LE (Cvekl and Tamm, 2004). It was reasoned that the observed elevation of mTORC1 signalling (Fig. S4B,C) in one or combinations of these tissues underlay the anterior segment phenotype observed in *Lhx2-Cre:Tsc1^f/f^* mice. The distribution of pS6 was therefore assessed within these domains during embryogenesis by immunohistochemistry (Fig. 4A-P). No discernible differences were observed in the levels and distribution of pS6 in the optic cup, the overlying POM or the LE at E10.5 (Fig. 4A,E,I,M). By contrast, a premature increase in both pS6^S235/236^ and pS6^S240/244^ was detected within the CM, POM and LE of *Lhx2-Cre:Tsc1^f/f^* mice at E12.5 (Fig. 4B,F,J,N). At later embryonic stages, the levels of pS6^S235/236^ and pS6^S240/244^ continued to be elevated in the CM of mutant mice, whereas levels within the POM and LE appeared to be similar to that observed in control littermates (Fig. 4C,D,G,H,K,L,O,P).

Lineage-tracing experiments demonstrated that *Lhx2-Cre* transgene expression and consequent *Tsc1* ablation was solely restricted to the CM (Fig. 1B). The premature elevation of S6 phosphorylation within the CM of *Lhx2-Cre:Tsc1^f/f^* mice at E12.5 was therefore to be expected. However, as pS6 levels were also increased within the POM and LE of mutant animals, this suggests that aberrant paracrine signal(s) originating from the CM initiate a premature increase in mTORC1 signalling within these neighbouring domains (Fig. 4F,N). One pathway that can act in this paracrine manner during anterior segment development is retinoic acid (RA) signalling (Duester, 2009). The distribution of retinaldehyde dehydrogenase (*Raldh*) transcripts was therefore determined to demarcate areas of active RA synthesis within the developing eye (Cvekl and Wang, 2009; Duester, 2009; Matt et al., 2005; Molotkov et al., 2006). No discernible differences in the level and distribution of either *Raldh3* or *Raldh1* transcripts were observed within control and *Lhx2-Cre:Tsc1^f/f^* mice at E12.5 (Fig. S5A,B,D,E). *Raldh3* transcripts were enriched in the ventral retina but also within the dorsal CM and PC (Fig. S5A,D) whereas *Raldh1* was highly expressed in the dorsal retina, the ventral CM and the LE (Fig. S5B,E) (Matt et al., 2005). Moreover, there was also no difference in Raldh1 protein expression within the dorsal retina of control and *Lhx2-Cre:Tsc1^f/f^* mice (Fig. S5C,F).

Taken together, these combined analyses demonstrate that conditional deletion of *Tsc1* during eye development leads to a reduction in hamartin levels and a subsequent premature increase in mTORC1 signalling within the CM of *Lhx2-Cre:Tsc1^f/f^* mice at E12.5. This sustained mTORC1 activity, in turn, induces mTORC1 signalling within the neighbouring POM and LE. However, non-autonomous phosphorylation of S6 within the POM and LE of *Lhx2-Cre:Tsc1^f/f^* mice does not appear to be mediated by paracrine RA signalling originating from the CM.

### Conditional deletion of *Tsc1* alters transcriptional programs within the ciliary margin

**Fig. 5.**
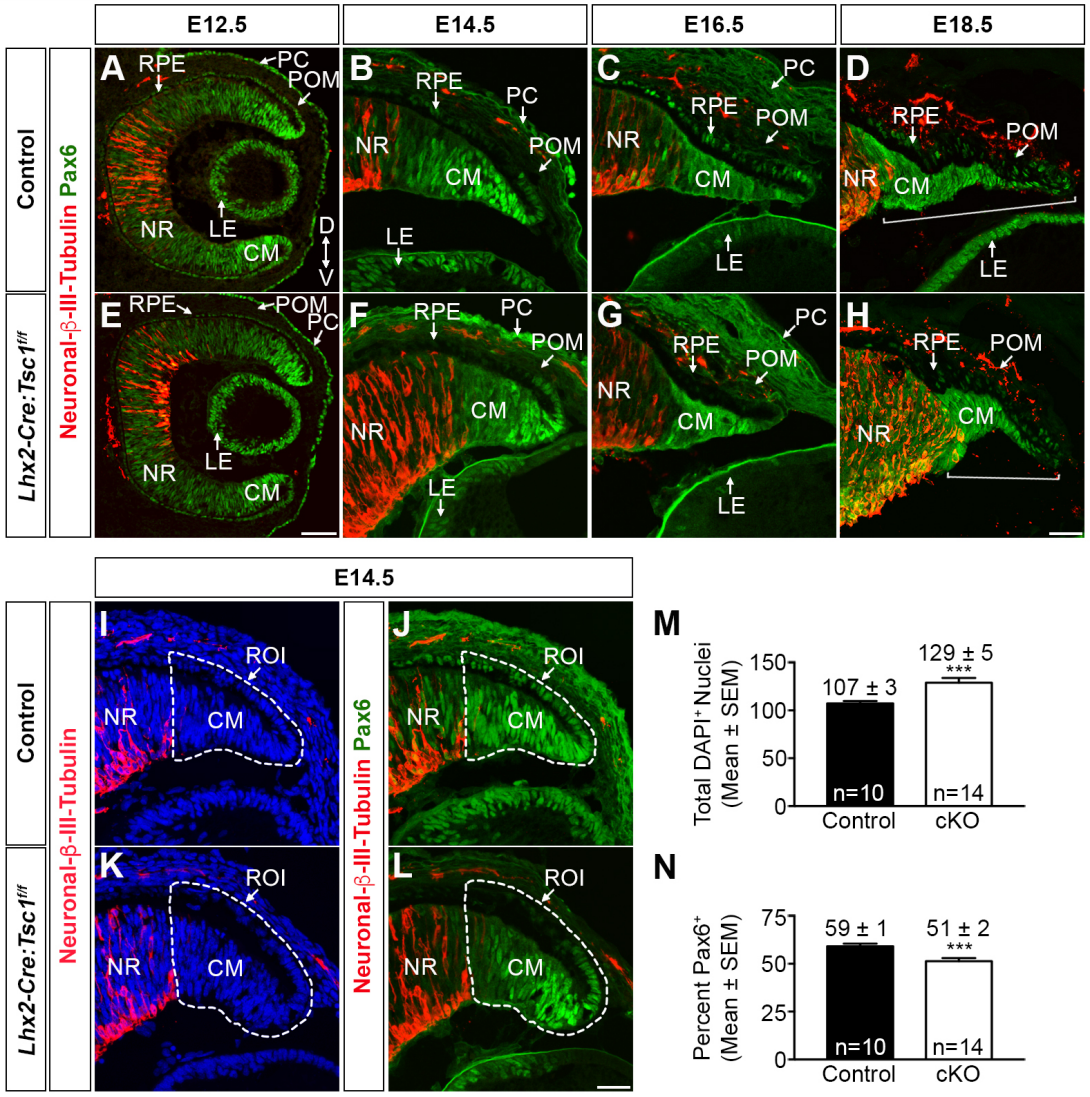
**Conditional deletion of *Tsc1* leads to a reduction in the proportion of Pax6^+^ cells within the ciliary margin.** (A-H) Immunohistochemical analysis of coronal eye sections taken from control (A-D) and *Lhx2-Cre:Tsc1^f/f^* (E-H) mice at E12.5 (A,E), E14.5 (B,F), E16.5 (C,G) and E18.5 (D,H) demonstrates that although a comparable central^low^ to distal^high^ gradient of Pax6 protein distribution is observed at all ages, there is an apparent reduction in the proportion of Pax6^+^ cells within the CM of mutant animals. A reduction in total CM length is also observed in *Lhx2-Cre:Tsc1^f/f^* mice at E18.5 (D,H, brackets) when compared with control animals. CM length is defined as beginning at the distal tip of the CM to the initiation of neuronal β-III-tubulin staining. Note that overlapping neuronal β-III-tubulin and Pax6 immunoreactivity within the NR demarcates developing amacrine and retinal ganglion cells (Ma et al., 2007). (I-N) Quantification of the number of Pax6^+^ cells in the CM of control and *Lhx2-Cre:Tsc1^f/f^* mice at E14.5 using immunohistochemical analysis of Pax6 and neuronal β-III-tubulin. A region of interest (ROI) was drawn around the presumptive CM (I,K, broken line) and the number of DAPI^+^ cells was then quantified within this ROI. We observed that *Lhx2-Cre:Tsc1^f/f^* mice (cKO) had a significant increase in the number of DAPI^+^ cells within the CM at E14.5 when compared with control littermates (M). The number of Pax6^+^ cells was then quantified within the ROI (J,L). This Pax6 data was subsequently divided by the DAPI counts to determine the percentage of Pax6^+^ cells. We observed that *Lhx2-Cre:Tsc1^f/f^* mice (cKO) had a significant reduction in the percentage of Pax6^+^ cells at E14.5 when compared with control littermates (N). All quantification data represent mean±s.e.m. (*n*=10 eyes for control and *n*=14 eyes for *Lhx2-Cre:Tsc1^f/f^*). ***P≤0.001, calculated using an unpaired two-tailed Student's *t*-test. Scale bars: 50 µm (A,E); 25 µm (B-D,F-H,I-L).

Aberrant mTORC1 signalling disturbs molecular homeostasis and this underlies a variety of pathological conditions (Duvel et al., 2010). Alterations in the level and/or pattern of gene expression within the CM, POM and/or LE upon premature mTORC1 activation could underlie the CB and iris phenotype observed in *Lhx2-Cre:Tsc1^f/f^* mice. Initial focus was given to genes whose expression is located predominantly within the CM since *Lhx2-Cre* transgene expression and consequent *Tsc1* ablation was restricted to this domain (Fig. 1B). Anterior segment development is completely reliant on the correct *Pax6* gene dosage (Davis et al., 2009; Davis-Silberman et al., 2005; Hill et al., 1991). The levels and distribution of Pax6 within the CM of control and *Lhx2-Cre:Tsc1^f/f^* mice was therefore assessed in combination with neuronal β-III-tubulin (Tubb3) to demarcate the CM and neural retina (NR) (Fig. 5). A comparable central^low^ to distal^high^ gradient of Pax6 distribution was established in both control and *Lhx2-Cre:Tsc1^f/f^* mice at E12.5, with the highest levels detected within the CM (Fig. 5A,E). At later embryonic ages, the Pax6 gradient was maintained, although there was an apparent reduction in the proportion of Pax6^+^ cells within the CM of mutant animals at all ages analysed (Fig. 5B-D,F-H). The number of Pax6^+^ cells within the CM was therefore quantified to address this apparent reduction. A significant decrease (−14%) in the number of Pax6^+^ cells was observed in the CM of *Lhx2-Cre:Tsc1^f/f^* mice at E14.5 when compared with controls (Fig. 5I-N). In addition, the CM length, as defined by the absence of neuronal β-III-tubulin expression was consistently shorter in *Lhx2-Cre:Tsc1^f/f^* mice at E18.5 (Fig. 5D,H, brackets).

**Fig. 6.**
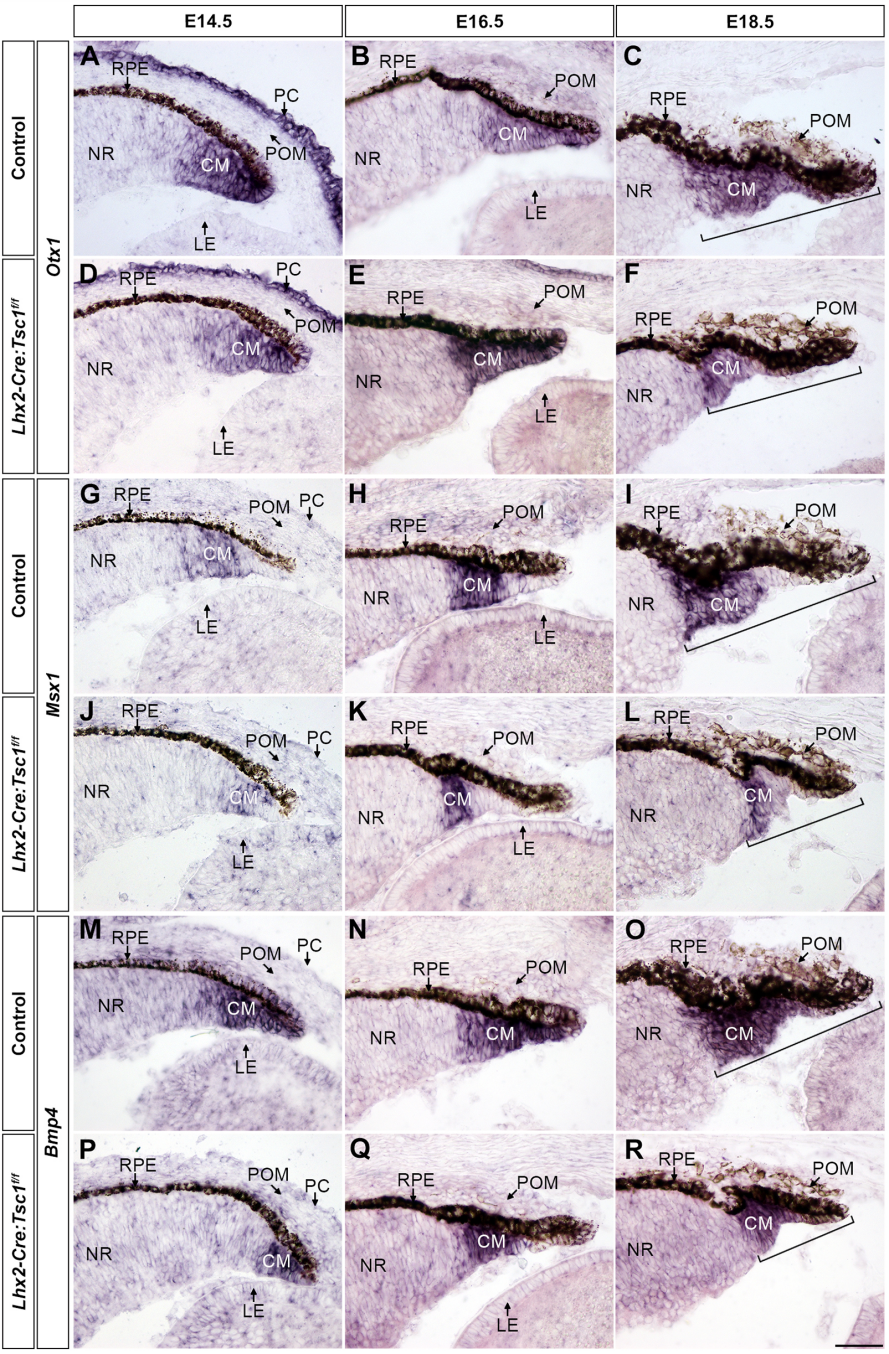
**Conditional deletion of *Tsc1* disrupts transcriptional programs within the ciliary margin.** (A-F) *In situ* hybridisation analysis of coronal eye sections taken from control (A-C) and *Lhx2-Cre:Tsc1^f/f^* (D-F) mice at E14.5 (A,D), E16.5 (B,E) and E18.5 (C,F) demonstrates a comparable expression of *Otx1* within the CM at E14.5 (A,D) and E16.5 (B,E). Also note the expression of *Otx1* in the overlying PC at E14.5 (A,D). (G-L) *In situ* hybridisation analysis of coronal eye sections taken from control (G-I) and *Lhx2-Cre:Tsc1^f/f^* (J-L) mice at E14.5 (G,J), E16.5 (H,K) and E18.5 (I,L) demonstrates a reduction in the number of *Msx1*-expressing cells within the CM of mutant animals at all ages analysed. (M-R) *In situ* hybridisation analysis of coronal eye sections taken from control (M-O) and *Lhx2-Cre:Tsc1^f/f^* (P-R) mice at E14.5 (M,P), E16.5 (N,Q) and E18.5 (O,R) shows a reduction in the number of cells that express *Bmp4* within the CM of mutant animals at E14.5 (M,P). No difference in *Bmp4* expression is seen at E16.5 (N,Q). In addition, a reduction in total CM length at E18.5 is consistently observed in *Lhx2-Cre:Tsc1^f/f^* mice when compared with control animals. CM length is defined as beginning from the distal tip of the CM to the proximal part of the *Otx1*, *Msx1* and *Bmp4* expression domains (C,F,I,L,O,R, brackets). Scale bar: 50 µm (A-R).

The expression of additional marker genes implicated in CB and iris development was subsequently assessed by *in situ* hybridisation to independently verify the reduction in CM progenitor cells in *Lhx2-Cre:Tsc1^f/f^* mice (Fig. 6). A comparable spatial distribution of *Otx1* transcripts (Martinez-Morales et al., 2001) was observed within the CM of both control and mutant animals at E14.5 and E16.5 (Fig. 6A,B,D,E). However, a reduction in the number of cells expressing the presumptive CB marker msh homeobox 1 (*Msx1*) (Monaghan et al., 1991) was detected in *Lhx2-Cre:Tsc1^f/f^* mice at all ages analysed (Fig. 6G-L). Bone morphogenetic protein (BMP) signalling is required for CB morphogenesis in part by modulation of *Msx1* expression (Chang et al., 2001; Zhao et al., 2002). Accordingly, a reduction in the number of cells that express *Bmp4* was also observed at E14.5 within the CM of mutant animals when compared with littermate controls (Fig. 6M,P), but no discernible difference in the number of *Bmp4*-expressing cells was seen at E16.5 (Fig. 6N,Q). Moreover, it was also confirmed that CM length, as defined by the transcriptional domains for all genes, was always consistently shorter in *Lhx2-Cre:Tsc1^f/f^* mice at E18.5 (Fig. 6C,F,I,L,O,R and Fig. S6, brackets).

Premature mTORC1 signalling was also observed within the POM and LE of *Lhx2-Cre:Tsc1^f/f^* mice (Fig. 4F,N) We therefore assessed whether aberrant gene networks within these domains also contributed to the CB and iris phenotype observed in mutant animals. Particular focus was given to genes that are predominantly expressed in the POM and LE, and are implicated in ASD and/or signalling pathways involved in anterior segment development. Both *Pitx2* and *Foxc1* are established ASD candidate genes, whereas Wnt signalling orchestrates CB and iris development (Gage et al., 2008; Lines et al., 2002; Liu et al., 2007). The expression patterns of *Pitx2*, *Foxc1* and *Wnt2b* in addition to the Wnt signalling components *Dkk2* (dickkopf Wnt signalling pathway inhibitor 2) and *Axin2* were therefore analysed. No differences in transcript distribution within the POM or LE were observed for any of these genes (Figs S7 and S8).

Taken together, these data demonstrate that conditional deletion of *Tsc1* during eye development leads to a reduction in the proportion of Pax6^+^ cells in the CM. This, in combination with a decrease in the number of cells that express *Bmp4* and *Msx1*, presumably leads to the consistent reduction in overall CM length observed at E18.5. Moreover, it appears that CM cell-autonomous mechanisms predominantly underlie the CB and iris phenotype observed in postnatal *Lhx2-Cre:Tsc1^f/f^* mice since no changes in the transcriptional distribution of selected candidate genes was observed within the POM and LE.

### Conditional deletion of *Tsc1* disrupts postnatal ciliary body and iris development

**Fig. 7.**
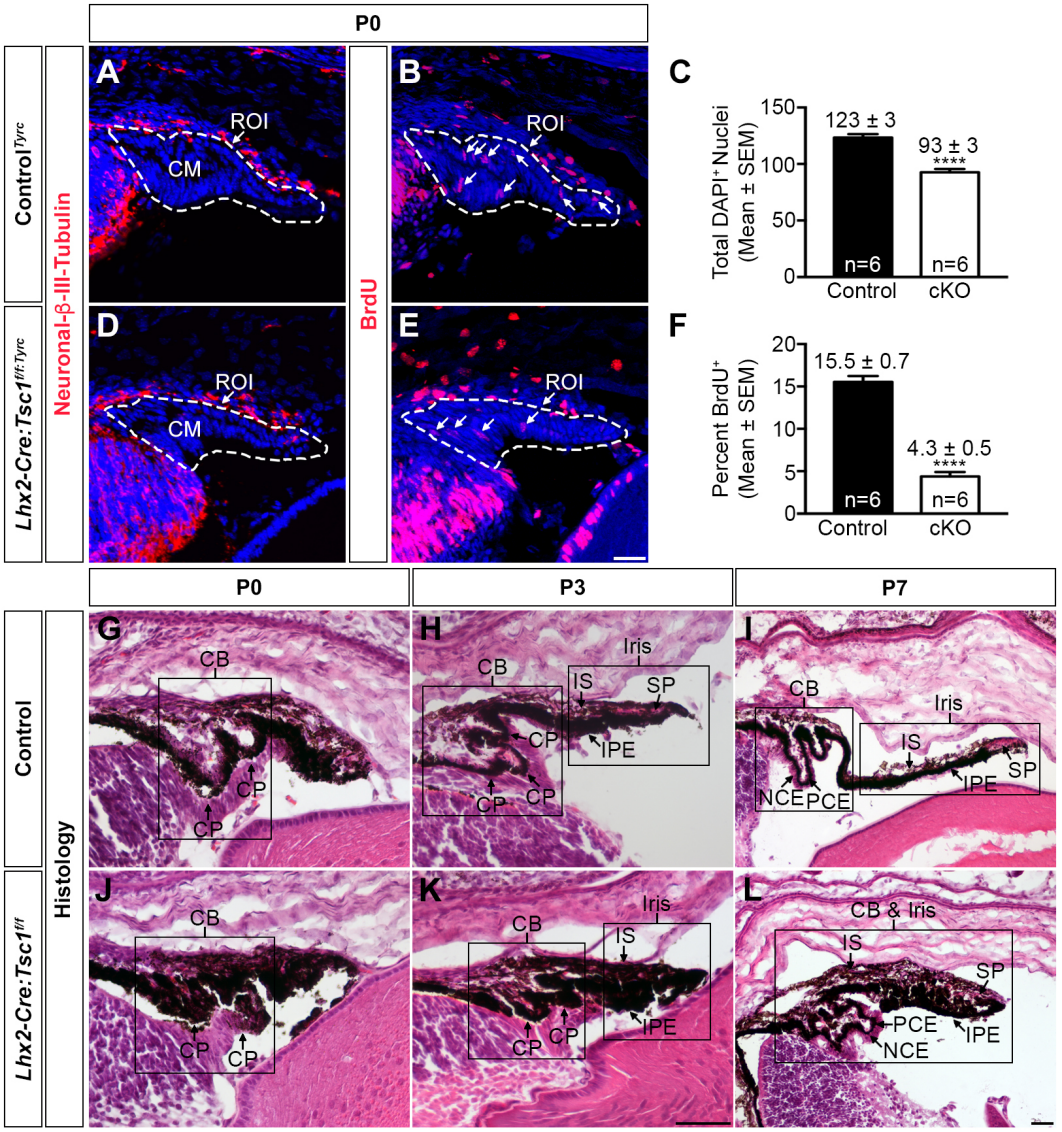
**Conditional deletion of *Tsc1* disrupts postnatal ciliary body and iris development.** (A-F) CM proliferation rate analysis on coronal eye sections taken from control*^Tyrc^* (A,B) and *Lhx2-Cre:Tsc1^f/f:Tyrc^* (D,E) mice at P0. Immunohistochemical analysis for neuronal β-III-tubulin was used to define the NR and an ROI was drawn around the presumptive CM (A,D, dashed line). The number of DAPI^+^ cells was then quantified within this ROI. *Lhx2-Cre:Tsc1^f/f:Tyrc^* mice (cKO) had a significant reduction in the total number of DAPI^+^ cells within the CM at P0 when compared with control littermates (C). Immunohistochemical analysis for BrdU was performed on adjacent sections and the previously drawn ROI was used to define the CM (B,E, dashed line). The number of BrdU^+^ cells was then quantified within this ROI. This data was subsequently divided by the DAPI^+^ counts to determine the percentage of BrdU^+^ cells within the ROI. *Lhx2-Cre:Tsc1^f/f:Tyrc^* mice (cKO) had a significant reduction in the percentage of proliferating cells in the CM at P0 when compared with control*^Tyrc^* littermates (F). All quantification data are mean±s.e.m. (*n*=6 eyes for both control*^Tyrc^* and *Lhx2-Cre:Tsc1^f/f:Tyrc^*); ****P≤0.0001, calculated using an unpaired two-tailed Student's *t*-test. (G-L) Histological analysis of coronal eye sections taken from control (G-I) and *Lhx2-Cre:Tsc1^f/f^* (J-L) mice at P0 (G,J), P3 (H,K) and P7 (I,L). The CB and iris undergo gradual morphogenesis in control animals during the first postnatal week (G-I). The future CB is initially demarcated by the emergence of the ciliary processes at P0 (G). Further development of the ciliary processes coupled with IS and IPE extension in addition to the appearance of the SP is observed at P3 (H) and by P7, the overall structure of both the CB and iris are well defined (I). In contrast, the CB and iris fail to undergo morphogenesis during the first postnatal week in *Lhx2-Cre:Tsc1^f/f^* mice (J-L). This is best illustrated by the absence of well-defined ciliary processes at P0 (J) coupled with a failure of iris extension and SP development at P3 (K). This failure of morphogenesis eventually culminates in the hypotrophic appearance of both the CB and iris at P7 (L). Scale bars: 25 µm (A,B,D,E), 100 µm (G,H,J,K) and 200 µm (I,L).

Previous studies have demonstrated that decreases in Pax6 levels reduce the proliferation of CM cells (Davis et al., 2009; Davis-Silberman et al., 2005). Since a reduced number of Pax6^+^ cells was observed within the CM of *Lhx2-Cre:Tsc1^f/f^* mice (Fig. 5N), it was reasoned that one contributing factor to the failure of CB and iris morphogenesis in postnatal animals was a reduced rate of proliferation within the CM. Late stage embryos (E18.5) were therefore labelled with BrdU and label incorporation was determined at P0. The number of nuclei within the CM of control*^Tyrc^* and *Lhx2-Cre:Tsc1^f/f:Tyrc^* mice was first quantified and mutant animals were found to contain 24% fewer DAPI^+^ cells within this domain (Fig. 7A,C,D). However, the CM of mutant animals also demonstrated a 72% reduction in the number of BrdU^+^ cells, thus demonstrating a reduced rate of progenitor cell proliferation upon *Tsc1* ablation (Fig. 7B,E,F).

CB and iris morphogenesis occurs during the first postnatal week of mouse development (Napier and Kidson, 2007). Given that a decrease in overall CM length and progenitor proliferation was observed in *Lhx2-Cre:Tsc1^f/f^* mice it was next assessed how this reduction influenced CB and iris development. Histological analysis of sagittal eye sections taken from control mice demonstrated that the future CB was initially demarcated by the emergence of the ciliary processes at P0 (Fig. 7G) and by the completion of the first postnatal week they appeared as distinct foliated structures (Fig. 7H,I). Simultaneously, the iris elongated toward the centre of the lens and the SP became apparent (Fig. 7I). Thus, by P7, the overall structure of both the CB and iris were well established in control animals (Fig. 7G-I). In contrast, the CB and iris in *Lhx2-Cre:Tsc1^f/f^* mice failed to undergo morphogenesis, as illustrated by the absence of ciliary processes coupled with a lack of iris extension (Fig. 7J,K). Failure to reach these developmental milestones at P0 and P3 eventually culminated in the hypotrophic appearance of both the CB and iris at P7 (Fig. 7L).

Taken together, these data demonstrate that conditional deletion of *Tsc1* during eye development leads to a reduced number of progenitor cells within the CM of *Lhx2-Cre:Tsc1^f/f^* mice, with the remaining cells exhibiting a decreased rate of proliferation. This reduced proliferation rate subsequently leads to a failure of CB and iris morphogenesis during the first postnatal week in mutant animals and eventually culminates in the ASD phenotype observed in *Lhx2-Cre:Tsc1^f/f^* mice.

## DISCUSSION

**Fig. 8.**
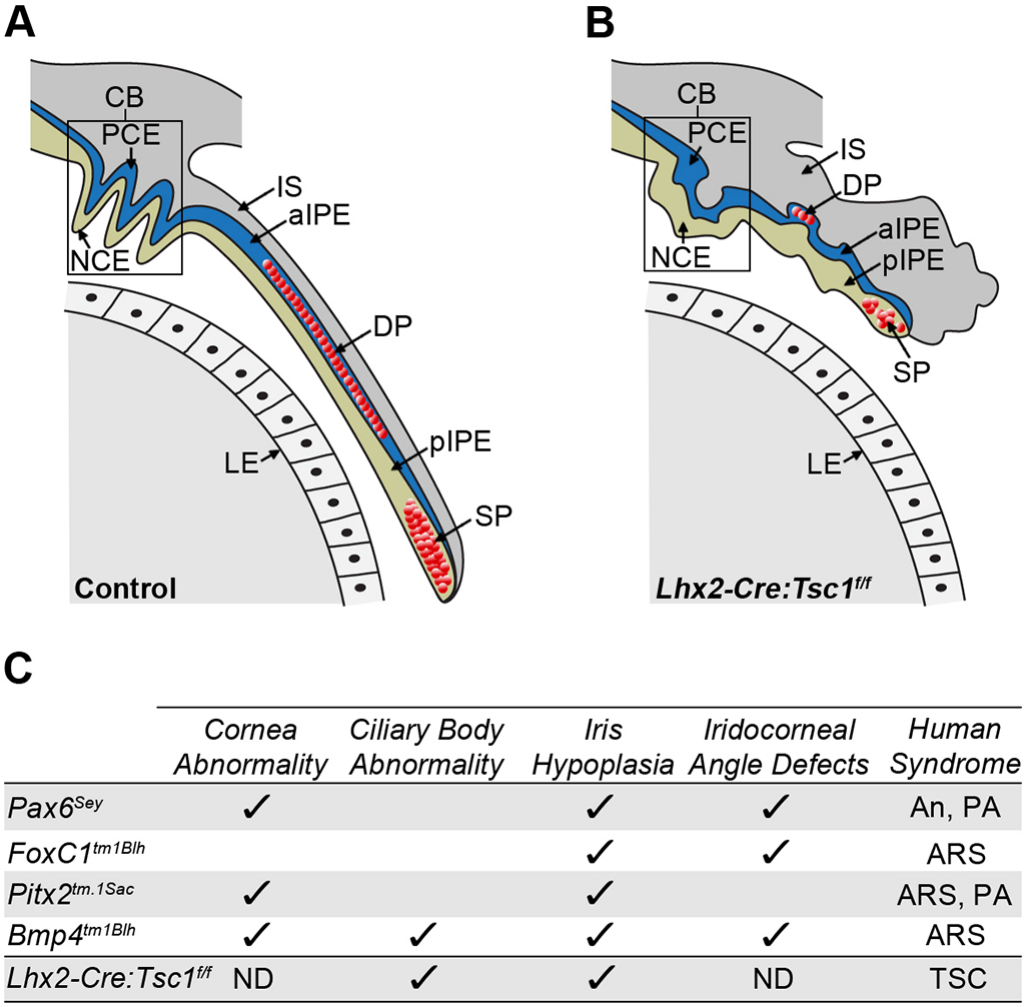
**Summary of the anterior segment phenotypes observed in *Lhx2-Cre:Tsc1^f/f^* mice and comparison to established mouse models of ASD.** (A) Control mice exhibit a CB with well-defined ciliary processes. Iris extension follows the curvature of the lens with the medial region containing the DP and the distal tip containing the SP. (B) *Lhx2-Cre:Tsc1^f/f^* mice exhibit hypotrophy of anterior eye structures. While the NCE and PCE of the CB are both present, the overall structure lacks the presence of well-defined ciliary processes. Moreover, the iris fails to extend and presents as a hypotrophic structure with DP and SP atrophy clearly evident. (C) Comparison of anterior segment phenotypes observed in *Lhx2-Cre:Tsc1^f/f^* mice with selected phenotypes documented in established mouse models of ASD: *Pax6^Sey^* (Hill et al., 1991), *Foxc1^tm1Blh^* (Kume et al., 1998), *Pitx2^tm.1Sac^* (Gage et al., 1999) and *Bmp4^tm1Blh^* (Chang et al., 2001). aIPE, anterior iris pigment epithelium; An, aniridia; ARS, Axenfeld–Rieger syndrome; ND, not determined; PA, Peter's anomaly; pIPE, posterior iris pigment epithelium; TSC, tuberous sclerosis complex.

This study characterises a novel mouse model of ASD. Conditional deletion of *Tsc1* during eye development leads to a premature upregulation of mTORC1 activity within tissue domains whose interplay underlies anterior segment morphogenesis. This aberrant mTORC1 signalling leads to cell-autonomous alterations in genetic networks within the CM and a decrease in cell proliferation with consequent CB and iris hypoplasia in postnatal animals (Fig. 8A,B). Further characterisation of the *Lhx2-Cre:Tsc1^f/f^* mice is required in order to precisely define which particular ASD syndrome the model best represents. These studies could encompass histological and molecular analyses of the lens, cornea and associated drainage structures since aberrant development of these anterior eye tissues also contributes to ASD presentation (Idrees et al., 2006; Ito and Walter, 2014). But the neuroectodermal origin of the affected genetic networks within the CM and consequent anterior segment phenotype observed in *Lhx2-Cre:Tsc1^f/f^* mice is strikingly similar to the underlying molecular aetiology and clinical presentation documented for individuals with aniridia (Beauchamp and Meisler, 1986; Lee et al., 2008). Moreover, as some TSC patients present with anterior segment deficiencies such as aniridia, this suggests that one facet of this systemic syndrome involves ASD in some affected individuals (Eagle et al., 2000; Gutman et al., 1982; Kranias and Romano, 1977; Lucchese and Goldberg, 1981; Milea and Burillon, 1997; Welge-Lussen and Latta, 1976). Given these corroborative experimental and clinical observations, we therefore propose that the *Lhx2-Cre:Tsc1^f/f^* mice described in this study should be added to the growing list of established ASD models (Fig. 8C) (Gould and John, 2002).

Pax6 is a master regulator of eye morphogenesis (Gehring and Ikeo, 1999). This is best exemplified by point mutations in the human *PAX6* gene leading to aniridia (Lee et al., 2008), while modulation of mouse Pax6 levels reduces cell proliferation and leads to CB and iris hypoplasia (Davis et al., 2009; Davis-Silberman et al., 2005; Lee et al., 2008; Marquardt et al., 2001). The reduced number of proliferative Pax6^+^ cells residing in the CM during embryogenesis is therefore presumably the main driving mechanism underlying the anterior segment phenotype observed in postnatal *Lhx2-Cre:Tsc1^f/f^* mice. However, the precise molecular mechanism by which aberrant mTORC1 activity induces this reduction in CM progenitor cell number remains to be determined. But these findings do concur with recent reports where conditional deletion of *Tsc1* or *Tsc2* results in a decrease in the number of progenitor cells and consequent tissue hypoplasia during mammary gland and lung development (Qin et al., 2016; Ren et al., 2016). It is also interesting to note that contrasting segment-specific phenotypes exist within *Lhx2-Cre:Tsc1^f/f^* mice. A previous report demonstrated that neuroectodermal *Tsc1* ablation leads to retinal hyperplasia and corroborates the effects of sustained mTORC1 signalling in other TSC-affected organs (Jones et al., 2015) whereas we show here that conditional deletion of *Tsc1* within the same progenitor pool leads to hypoplasia within the anterior segment. Why contrasting compartment-specific developmental deficits exist within the eye of *Lhx2-Cre:Tsc1^f/f^* mice is therefore an intriguing avenue for future investigation. However, the CB hypoplasia seen in *Lhx2-Cre:Tsc1^f/f^* mice cannot merely be attributed to a reduction in the number of Pax6^+^ cells alone: other transcription factors and/or signalling molecules probably contribute to this particular phenotype. Possible candidates that fulfil this role are *Bmp4* and *Msx1* since both of these genes are indispensable for anterior segment morphogenesis (Chang et al., 2001; Zhao et al., 2002). That a reduced number of cells expressing *Bmp4* and *Msx1* was detected in the CM of *Lhx2-Cre:Tsc1^f/f^* mice therefore presumably contributes to the CB phenotype. However, it remains to be determined whether the observed CB hypoplasia involves interplay between Pax6, *Bmp4* and *Msx1* or whether these factors mediate separate developmental deficits.

The severity of the ASD phenotype produced by *Tsc1* ablation was influenced by genetic background. On a *129/SvCBAC57BL/6* mixed genetic background, the presentation of CB and iris hypoplasia was fully penetrant and consistent. This was in contrast to that seen in the *129/SvCBAC57BL/6NMRI* mixed genetic background, where the severity of ASD presentation was variable amongst littermates, albeit with full penetrance. These observations are in agreement with previous studies where allelic loss of *Foxc1*, *Bmp4* or *Col4a1* resulted in various anterior segment abnormalities with phenotype severity being entirely dependent upon genetic background (Chang et al., 2001; Mao et al., 2015; Smith et al., 2000). Taken together, our combined observations demonstrate that strain-specific modifier genes consistently alter the phenotype resulting from a particular ASD-causing mutation. Identification and characterisation of these modifier genes in different genetic backgrounds may therefore provide an important route for understanding the cellular and genetic interactions underlying the variations in ASD presentation observed in *Lhx2-Cre:Tsc1^f/f^* mice on the two mixed genetic backgrounds employed in this study. However, we cannot completely eliminate the possibility that unique stochastic developmental effects within individual embryos also contribute to the divergent spectrum of CB and iris phenotypes seen in these mutant mice (Gould and John, 2002). It is entirely possible that variations in the level of active mTORC1 signalling within individual embryos influence the temporal and spatial activation of anterior segment developmental genes. Appropriate activation of these regulators in some cells and incorrect and/or no activation in others may therefore contribute to the phenotypic variability observed in littermates on the *129/SvCBAC57BL/6NMRI* mixed genetic background.

Activation of the *ROSA26R* construct by *Lhx2-Cre*-mediated recombination precisely defined the contribution of optic cup progenitor cells to specific anterior segment structures. To the best of our knowledge, this lineage-tracing analysis is the first clear genetic demonstration that progenitor cells residing within the CM of the embryonic mouse eye contribute the epithelial layers of both the CB and iris in addition to the iridial muscles. These observations substantiate formative histological analyses of CB and iris development and confirm that species-specific differences exist during anterior segment development (Beebe, 1986; Imaizumi and Kuwabara, 1971). This is best exemplified by our results demonstrating that the mouse iridial muscles are derived from optic cup progenitor cells, whereas these structures originate from the neural crest in birds (Creuzet et al., 2005; Johnston et al., 1979; Nakano and Nakamura, 1985). The *Lhx2-Cre* transgene is therefore a powerful molecular tool that can be used to elucidate the genetic networks that underlie the development and disease of both posterior and anterior segment structures that arise from neuroectodermal progenitor cells. Moreover, combinatorial analyses using the *Lhx2-Cre* transgene in conjunction with other compatible lineage-tracing reporter systems would be an invaluable approach to gain a better understanding as to how cells originating from different embryonic regions simultaneously migrate to assemble the eye (Legué and Joyner, 2010).

To conclude, this study describes the characterisation of a novel mouse model of ASD. This model provides a valuable resource for future studies concerning the molecular mechanisms underlying ocular syndromes and also serves as a platform to evaluate new therapeutic approaches for the treatment of visual disorders. In addition, the combined studies on anterior and posterior segment development in *Lhx2-Cre:Tsc1^f/f^* mice (Jones et al., 2015) demonstrate that the various tissues contributing to the formation of the adult mouse eye require separate and distinct levels of mTORC1 signalling for correct morphogenesis.

## MATERIALS AND METHODS

### Animals

All animal experiments were approved by the animal review board at the Court of Appeal of Northern Norrland in Umeå. The derivation and genotyping of *Tg(Lhx2-Cre)1Lcar* transgenic mice (abbreviated to *Lhx2-Cre*), *Tsc1^tm1Djk^* floxed mice (*Tsc1^+/f^* or *Tsc1^f/f^*) and *Gt(ROSA)26Sor^tm1sor^* reporter mice (*ROSA26R*) have been described previously (Hägglund et al., 2011; Soriano, 1999; Uhlmann et al., 2002). The genotype of all animals was determined by PCR analysis of genomic DNA extracted from tail biopsies. Breeding *Lhx2-Cre:Tsc1^+/f^* and *Tsc1^f/f^* mice or *Lhx2-Cre* and *ROSA26R* mice generated all experimental animals. The morning of the vaginal plug was considered as E0.5. Both males and females were used for experimental analyses and littermates lacking the *Lhx2-Cre* transgene were used as controls. All analyses were carried out on a *129/SvCBAC57BL/6* (control and *Lhx2-Cre:Tsc1^f/f^*) and/or *129/SvCBAC57BL/6NMRI* mixed genetic backgrounds. The latter strain was generated by crossing *129/SvCBAC57BL/6* mice with *BomTac:NMRI* mice, which lack pigment due to a mutation in the *Tyr* gene (*Tyr^c^*). The *129/SvCBAC57BL/6NMRI* mixed background strain was then bred to be homozygous for the *Tyr^c^* mutation (control*^Tyrc^* and *Lhx2-Cre:Tsc1^f/f:Tyrc^*). All postnatal analyses of *Lhx2-Cre:Tsc1^f/f^* mice were conducted before P21 since this model dies from neurological complications at approximately 3 weeks of age (Jones et al., 2015).

### Histology

Heads or enucleated eyes were fixed in 2% (w/v) glutaraldehyde and 3% (w/v) paraformaldehyde (PFA) in PBS overnight at 4°C. Tissues were then either immersed in 70% (v/v) ethanol and paraffin embedded or equilibrated in 30% (w/v) sucrose in PBS overnight at 4°C and embedded in OCT compound (Sakura Finetek). Paraffin sections (10 µm) were subsequently cleared in xylene (2×5 min) before rehydration through a series of ethanol washes [99.5%, 95%, 90% and 80% (v/v) in PBS]. Cryosections (10 µm) were rehydrated in PBS (2×5 min). Haematoxylin and Eosin staining was performed as previously described (Hägglund et al., 2011).

### Lineage tracing

Heads or enucleated eyes were fixed in 4% (w/v) PFA in PBS for 30 min on ice and then equilibrated in 30% (w/v) sucrose in PBS overnight at 4°C. Tissues were embedded in OCT compound (Sakura Finetek) and cryosections (10 µm) were prepared. Lineage-tracing analyses were performed as described previously (Hägglund et al., 2011).

### Immunohistochemistry

Heads or enucleated eyes were fixed in 4% (w/v) PFA in PBS for up to 2 h on ice. The tissues were equilibrated overnight at 4°C in 30% (w/v) sucrose in PBS and embedded in OCT compound (Sakura Finetek). Immunohistochemistry was performed on cryosections (10 µm) as previously described (Ma et al., 2007). An additional blocking step involving MOM Blocking Reagent (Vector Labs) was used in all experiments involving monoclonal primary antibodies. The following antibodies and dilutions were used: αSMA (1:200, Abcam, ab5694 and 1:500, Sigma-Aldrich, C6198), β-gal (1:500, MP Biomedical, 0855976); Cav3 (1:500, BD Biosciences, 610421), neuronal β-III-tubulin (1:1000, Covance, MRB-435), Pax6 (1:100, DSHB, Clone P3U1), pS6^S235/236^ (1:100, Cell Signaling Technology, 4857), pS6^S240/244^ (1:100, Cell Signaling Technology, 5364) and Raldh1 (1:4000, Abcam, ab96060). All immunohistochemistry antibodies have been independently verified in previous studies (Chae et al., 2010; Ericson et al., 1997; Hägglund et al., 2013; Jones et al., 2015; Kern et al., 2013; Kogo et al., 2006; Miao et al., 2016).

### Immunoblotting

The lens was removed from enucleated eyes and the NR and RPE frozen in liquid nitrogen. Tissues were homogenised in SDS lysis buffer [100 mM Tris-HCl, pH 6.8; 2% (w/v) SDS] containing both protease and phosphatase inhibitors (Complete Mini & PhosSTOP, Roche) using a TissueLyser (Qiagen). The concentration of the soluble protein fraction was measured using the BCA Protein Assay Kit (Fisher Thermo Scientific). Immunoblotting was performed on protein extracts (6 µg) using Criterion TGX gels and nitrocellulose membranes (Bio-Rad) (Mahmood and Yang, 2012). Reactive proteins were visualised using SuperSignal West Dura Extended Duration Substrate (Fisher Thermo Scientific). Quantification was performed using a ChemiDoc MP Imaging System (Bio-Rad). The following antibodies and dilutions were used: Hamartin (1:1000, Cell Signaling Technology, 4906), S6 (1:2000, Cell Signaling Technology, 2217), pS6^S235/236^ (1:1000, Cell Signaling Technology, 4857), pS6^S240/244^ (1:2000, Cell Signaling Technology, 5364) and GAPDH (1:30,000, Cell Signaling Technology, 2118). All immunoblotting antibodies have been verified in a previous study (Jones et al., 2015).

### *In situ* hybridisation

Heads were fixed in 4% (w/v) PFA in PBS for 2 h on ice and equilibrated in 30% (w/v) sucrose in PBS overnight at 4°C. Tissues were embedded in OCT compound (Sakura Finetek). *In situ* hybridization on cryosections (10 µm) was performed as previously described (Schaeren-Wiemers and Gerfin-Moser, 1993). The following cDNA templates or IMAGE clone were used to generate riboprobes: *Axin2* (BC057338, nucleotide position 1-1520), *Bmp4* (NM_007554, nucleotide position 117-578), *Bmp7* (NM_007557, nucleotide position 1-1987), *Dkk2* (BC096448, nucleotide position 1-3713), *Foxc1* (NM_008592, nucleotide position 526-1354), *Msx1* (NM_010835, nucleotide position 274-1138), *Otx1* (NM_011023, nucleotide position 168-962), *Pitx2* (NM_001042504, nucleotide position 59-799), *Raldh1* (BC054386, nucleotide position 755-2078), *Raldh3* (NM_053080, nucleotide position 247-1088), *Rdh10* (NM_133832, nucleotide position 14-923), *Sfrp1* (NM_013834, nucleotide position 109-923), *Wnt2b* (IMAGE clone #40057540) and *Zic1* (NM_009573, nucleotide position 272-1176).

### Proliferation analyses

Pregnant dams were injected intraperitoneally at E18.5 with BrdU at 50 µg/g body weight and pups were collected at P0. Heads were fixed in 4% (w/v) PFA in PBS for 2 h on ice and then equilibrated overnight at 4°C in 30% (w/v) sucrose in PBS before embedding in OCT compound (Sakura Finetek). Cryosections (10 µm) were washed in PBS and then denatured in 2 M HCl for 30 min at 37°C. The slides were subsequently neutralised with 100 mM borate buffer (pH 8.5) for 10 min at RT and then processed for immunohistochemistry as previously described using an anti-BrdU antibody (1:20, BD Pharmingen, 560210) (Jones et al., 2015).

### Image analyses

Images were captured using either a Zeiss LSM 710 confocal microscope, a Nikon Eclipse E800 microscope fitted with a Nikon DS-Ri1 digital colour camera or a Nikon SMZ1500 microscope fitted with a Nikon D5200 digital colour camera. All images were compiled and analysed using Fiji (Schindelin et al., 2012), CellProfiler (Carpenter et al., 2006; Kamentsky et al., 2011), Adobe Photoshop and Adobe Illustrator.

### Statistical analyses

All statistical analyses were performed using Prism7 (GraphPad). Quantification analyses were performed blind to genotype and unpaired two-tailed Student's *t*-tests were used to determine statistical significances. Error bars in all figures represent s.e.m. *P*-values are indicated as *P≤0.05, **P≤0.01, ***P≤0.001 and ****P≤0.0001.
